# Characterization of the complete mitochondrial genome of the Meiren yak (*Bos grunniens*)

**DOI:** 10.1080/23802359.2020.1726222

**Published:** 2020-02-11

**Authors:** Xian Guo, Xiaoyun Wu, Pengjia Bao, Zhen Yang, Zhi Dang, Kelei He, Xiaoli Yang, Shengguang Shi, Jie Pei, Chunnian Liang

**Affiliations:** a Key Laboratory of Yak Breeding Engineering of Gansu Province, Lanzhou Institute of Husbandry and Pharmaceutical Sciences, Chinese Academy of Agricultural Sciences, Lanzhou, People’s Republic of China;; b Animal Husbandry Station of Hezuo City, Hezuo, People’s Republic of China

**Keywords:** Bayesian inference, high-throughput sequencing, iterative mapping, mitogenomics, yak breed

## Abstract

In this study, high-throughput Illumina sequencing was employed to assemble the complete mitochondrial genome of the Meiren yak (*Bos grunniens*), a local yak breed from Gansu Province, China. The mitochondrial genome is 16,321 bp long with an A + T-biased nucleotide composition and harbors 13 protein-coding, 22 Trna, and 2 rRNA genes, and a noncoding control region. The mitogenomic organization and codon usage are highly similar to those of previously published congeneric mitochondrial genomes. Bayesian phylogenetic analysis indicates that Meiren yak is most closely related to nine other yak breeds (incl. Datong, Huanhu, Pali, Pamir, Polled, Qilian, Seron, Sunan, and Tianjun yaks).

Domestic yaks (*Bos grunniens*) are widely maintained across the Qinghai-Tibetan Plateau and adjacent regions and provides various living necessities for local residents (Qiu et al. 2012). To date, tens of yak breeds adapted to local environments have been identified across its geographic range. Here, we present the complete mitochondrial genome for Meiren yak, a local yak breed from Gansu Province, China. The annotated mitogenomic sequence has been deposited into GenBank under the accession number MN883671.

A blood sample was collected from Hezuo City, Gannan Tibetan Autonomous Prefecture, Gansu Province, China (35°00′N, 103°03′E), with the voucher specimen held in the Key Laboratory of Yak Breeding Engineering of Gansu Province, Lanzhou Institute of Husbandry and Pharmaceutical Sciences (Lanzhou, Gansu Province, China). The genomic DNA coded as NO.20191106, which was extracted from Meiren yak, is stored at −80 °C (ultra deep-freeze refrigerator) in the sample storage room of our department. Total genomic DNA was isolated with the QIAamp DNA Blood Mini Kit (Qiagen, CA, USA). Library preparation and high-throughput sequencing with Illumina HiSeq X^TM^ Ten Sequencing System (Illumina, CA, USA) were conducted by Annoroad Gene Technology (Beijing, China). Mitogenome assembly was done with the program MITObim v1.9 (Hahn et al. 2013); the initial reference (GenBank accession: JQ692071) was previously published by Qiu et al. (2012). Mitogenome annotation was done by aligning with those previously reported mitochondrial genomes of yak breeds (Wu et al. 2016; Guo et al. 2019; Huang et al. 2019; Huang, Zhang, Fu, et al. 2020; Huang, Zhang, Wu, et al. 2020).

The mitochondrial genome of Meiren yak is 16,321 bp long with an A + T-biased nucleotide composition (33.7% A, 25.8% C, 13.2% G & 27.3% T; ‘light strand’), and harbors 13 protein-coding genes (PCGs), 22 tRNAs, 2 rRNAs and a noncoding control region. Its genomic organization and codon usage are highly similar to those previously published congeneric mitochondrial genomes (e.g. Hiendleder et al. 2008; Chu et al. 2016; Wu et al. 2018; Guo et al. 2019; Zhou et al. 2019). The 13 PCGs are initiated with ATA or ATG codons and are terminated with TAA, TAG, or the incomplete T codons. The 22 tRNAs range in size from 60 (*tRNA-Ser*^AGN^) to 75 bp (*tRNA-Leu*^UUR^) with a total length of 1509 bp. The two rRNAs are 957 bp (*12S rRNA*) and 1571 bp (*16S rRNA*) long, respectively. The control region is 891 bp long with an A + T content of 61.0%.

A Bayesian tree was reconstructed to investigate its relationship with 15 other yak breeds using the program MrBayes v3.1.1 (Ronquist and Huelsenbeck 2003) as implemented in TOPALi v2.5 (Milne et al. 2009) (Figure 1). A concatenated sequence of all 13 PCGs excluding stop codons were used for the phylogenetic analysis, and ‘HKY + I’ was employed as the best-fit nucleotide substitution model as suggested by TOPALi v2.5. Two *Bison* species, i.e. *Bison bison* (Douglas et al. 2011) and *Bison priscus* (Froese et al. 2017), were included as outgroup taxa. The result showed that all 16 yak breeds were clustered into two major groups and that Meiren yak was most closely related to nine other yak breeds (incl. Datong, Huanhu, Pali, Pamir, Polled, Qilian, Seron, Sunan and Tianjun yaks).

**Figure 1.**
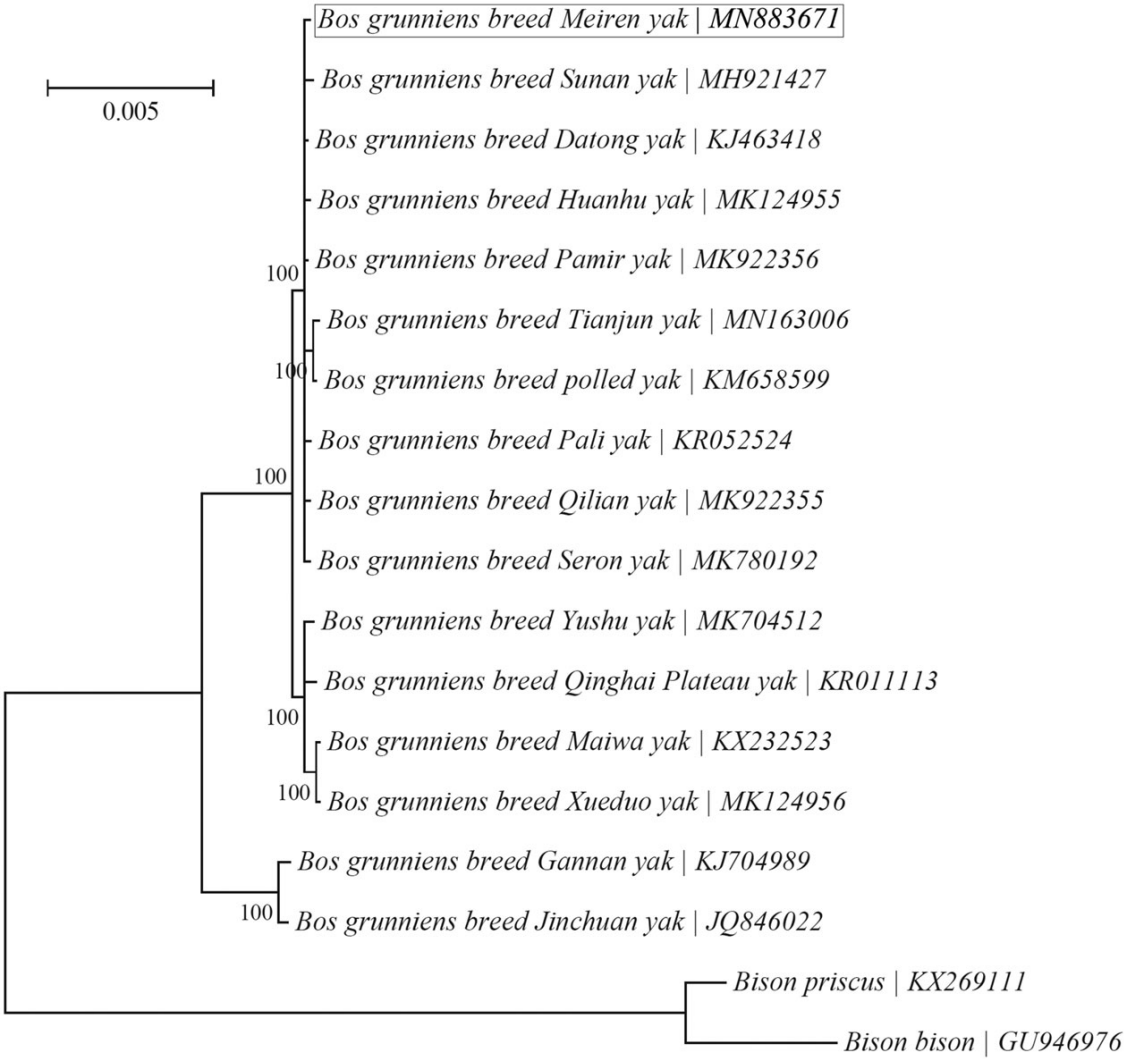
Phylogeny of 16 yak breeds based on the Bayesian analysis of the concatenated sequences of 13 mitochondrial protein-coding genes (alignment size: 11,370 bp). The best-fit nucleotide substitution model is ‘HKY + I’.
